# Screen-Printed Carbon Electrodes Modified with Cobalt Phthalocyanine for Selective Sulfur Detection in Cosmetic Products

**DOI:** 10.3390/ijms12063810

**Published:** 2011-06-09

**Authors:** Pei-Yen Chen, Chin-Hsiang Luo, Mei-Chin Chen, Feng-Jie Tsai, Nai-Fang Chang, Ying Shih

**Affiliations:** 1 Department of Cosmetic Science, Providence University 200 Chungchi Rd., Taichung 43301, Taiwan; E-Mails: pychen2@pu.edu.tw (P.-Y.C.); s9624065@pu.edu.tw (M.-C.C.); tftsay@pu.edu.tw (F.-J.T.); nfchang@pu.edu.tw (N.-F.C.); 2 Department of Safety, Health and Environmental Engineering, Hung Kuang University, 34 Chungchi Rd., Taichung 43302, Taiwan; E-Mail: andyluo@hk.edu.tw

**Keywords:** sulfur, cosmetics, cobalt phthalocyanine, screen-printed carbon electrode

## Abstract

Cobalt phthalocyanine (CoPc) films were deposited on the surface of a screen-printed carbon electrode using a simple drop coating method. The cyclic voltammogram of the resulting CoPc modified screen-printed electrode (CoPc/SPE) prepared under optimum conditions shows a well-behaved redox couple due to the (Co^I^/Co^II^) system. The CoPc/SPE surface demonstrates excellent electrochemical activity towards the oxidation of sulfur in a 0.01 mol·L^−1^ NaOH. A linear calibration curve with the detection limit (D_L_, S/N = 3) of 0.325 mg·L^−1^ was achieved by CoPc/SPE coupled with flow injection analysis of the sulfur concentration ranging from 4 to 1120 mg·L^−1^. The precision of the system response was evaluated (3.60% and 3.52% RSD for 12 repeated injections), in the range of 64 and 480 mg·L^−1^ sulfur. The applicability of the method was successfully demonstrated in a real sample analysis of sulfur in anti-acne creams, and good recovery was obtained. The CoPc/SPE displayed several advantages in sulfur determination including easy fabrication, high stability, and low cost.

## 1. Introduction

Sulfur is a naturally occurring element and significant for chemical, biological, and industrial applications. Sulfur constitutes an essential ingredient in various cosmetics, especially skin care products, as it is a keratolytic and germicidal agent, which improves acne, pimples, seborrhea and psoriasis [1]. Therefore, the US Food and Drug Administration (US FDA) allows the use of sulfur in over-the-counter (OTC) drugs for the treatment of acne and dandruff. Combined with other ingredients, 3% to 10% and 2% to 5% concentrations of sulfur are used in OTC anti-acne drugs and anti-dandruff shampoos, respectively [2]. However, sulfur is difficult to use in cosmetic products directly due to its poor solubility. To solve this problem, another sulfur compound (biosulfur fluid) was used. Biosulfur fluid is a sulfur and surfactant mixture used as a water-soluble sulfur source. The Association of Southeast Asian Nations (ASEAN) permits only 2% to 10% (w/w) of biosulfur fluid as part II ingredients (the substances cosmetic products must not contain, except subject to the restrictions and conditions laid down) in cosmetic products [3]. Since adverse effects manifest when sulfur limits are exceeded, it is essential to detect and determine the sulfur levels in cosmetic products. Numerous analytical methods such as high performance liquid chromatography (HPLC) [4,5], inductively coupled plasma (ICP) techniques [6–8], polarography [9,10], X-ray fluorescence [11] and gas chromatography (GC) [12–14] are available for measuring sulfur levels, but not in cosmetic products. Although, these techniques are accurate, fast, and sensitive, they are expensive and offer poor sulfur selectivity and sensitivity due to their complex sample preparation steps. In addition, simple and inexpensive indirect methods for determining sulfur as sulfate using ion chromatography (IC) [15,16], and titration [17,18] require tedious and time-consuming sample preparations. An electrochemical method is an attractive potential technique due to its low cost, convenience, and simplicity for direct sulfur assay. Since sulfur is electro-inactive, various modified electrodes, such as copper-mercury (Cu-Hg) amalgam and silver-mercury (Ag-Hg) amalgam electrodes with stripping voltammetric have been used for sulfur determination with good sensitivity (14 ng·L^−1^ in Cu-Hg and 4 ng·L^−1^ in Ag-Hg) [19]. However, the use of mercury amalgam always produces harmful mercury as a secondary environment pollutant. In a recent report, ion-selective electrodes were used to directly determine sulfur levels in cosmetic and pharmaceutical products, without any prior preparation methods [20,21]. This method was more precise with a low detection limit (0.04 mg·L^−1^) and superior than IC (24 mg·L^−1^) [16], however, the cost of the electrode and tedious sample preparation using toxic hydrazine hydrates are serious drawbacks in practice [20]. Therefore, an easy, direct, simple, cheap, rapid and cleaner method for the electrochemical determination of sulfur in cosmetics has merited continued research and development attention in recent years. Phthalocyanine complexes of transition metals, especially cobalt phthalocyanine (CoPc), are large macrocyclic molecules with applications in the fields of conductive polymers, chemical sensors, electrochromism, *etc*., due to their low cost facileness of large-scale preparation, and chemical and thermal stability. Numerous research groups have considered CoPc modified electrodes for determining sulfur containing compounds such as carbaryl [22], ethylene bisdithiocarbamate-based pesticides [23], cystine [24], and zinc pyrithione [25]. However, CoPc modified electrodes were rarely utilized for cosmetic product analysis. Flow injection analysis is a classic technique for precise and rapid sample analysis based on a high sensitivity, and low detection limit, and has a good linear range compared to other electrochemical techniques. This study used disposable screen- printed electrodes (SPE) modified with CoPc, (designated as CoPc/SPE) which successfully demonstrated a selective determination of sulfur in cosmetic products without any pretreatment processes using flow injection analysis (FIA).

## 2. Results and Discussion

### 2.1. Basic Electrochemical Studies

**Figure 1:**
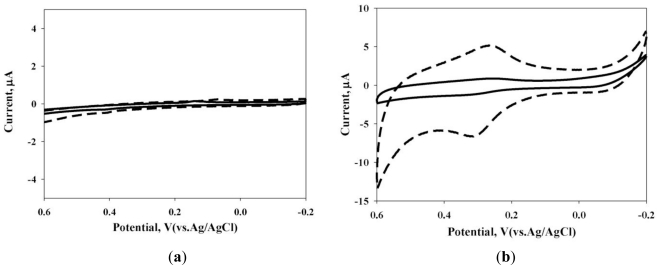
Cyclic voltammograms of SPE (**a**) and CoPc/SPE (containing 180 μg CoPc) (**b**) electrode with (dotted line) and without (solid line) 64 mg·L^−1^ of sulfur in a 0.01 mol·L^−1^ NaOH solution at sweep potential range −0.2 V to 0.6 V, *v* = 50 mV·s^−1^.

**Figure 2:**
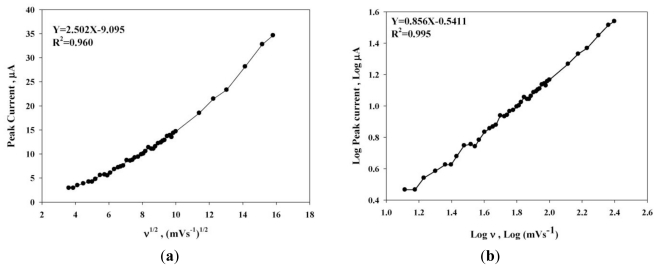
Typical plots of anodic peak current (*i_pa_*) *versus v*^1/2^ (**a**) and a double logarithmic of the anodic peak current (*i_pa_*) *versus v* (**b**).

Figure 1 shows cyclic voltammograms obtained at the CoPc/SPE electrode (containing 180 μg CoPc), with and without 64 mg·L^−1^ of sulfur in a 0.01 mol·L^−1^ NaOH solution, and at a scan rate (*v*) 50 mVs^−1^. The results obtained from bare SPE were also given in Figure 1 for comparison. In sulfur containing a NaOH solution, the cyclic voltammetric response of bare SPE was featureless (Figure 1a), while in the presence of a CoPc modified SPE, a reproducible and well defined faradic response to sulfur was obtained at *E_pa_* *−*0.30 V and *E_pc_* *−*0.27 V (*vs.* Ag/AgCl) (Figure 1b) in the potential range of *−*0.2 V to 0.6 V. During the transfer of Co^I^ to Co^II^ and Co^II^ to Co^I^ for the cathodic and anodic peak, similar electrochemcal behavior was reported [26]. This observation clearly demonstrates an electro-catalytic mediation of the CoPc towards the sulfur oxidation reaction. The peak separation potential, Δ*E_p_* (= *E_pa_* *– E_pc_*), is less than the (59/n) mV expected for a reversible system. Therefore, a CoPc redox couple at the CoPc/SPE demonstrates reversible behavior in an aqueous medium. CoPc/SPE film exhibits a high electrocatalytic efficiency, characterized by: (1) the decrease in overpotential for sulfur oxidation (no catalytic current was displayed in Figure 1a); (2) the electrocatalytic efficiency (5.8; estimated as the ratio (5.8; estimated as the ratio *i* _peak[S^2–^]=64mgL^–1^_ –*i*_peak[S^2–^]=0_ / *i*_peak[S^2–^]=0, at an applied potential of +310mV_) [27].

The values for the surface concentration (Γ) of CoPc/SPE, given in mol·cm^−2^, were obtained from the integrated charges (Q) of the anodic peak as follows:

$$\Gamma_{\text{CoPc}}=\text{Q}/n\text{FA}$$

where Q is the integrated charge of the CoPc/SPE anodic peak area corrected for the baseline, and easily observed in cyclic voltammograms; *n* is the number of electrons exchanged per reactant molecule (*n* = 1). The estimated surface concentration of CoPc/SPE was 1.94 × 10^−10^ mol·cm^−2^. The relationship between the sulfur oxidation current (*i^pa^*) and *v*^1^*^/^*^2^ in the electrocatalytic oxidation of sulfur at CoPc/SPE was further investigated. The sulfur oxidation current (*i_pa_*) was found to increase in correlation to a change in *v* from 13 to 250 mV·s^−1^. The sulfur oxidation current (*i_pa_*) *versus* f *v*^1/2^ (Figure 2a) and the double logarithmic plot of the sulfur oxidation current (*i_pa_*) *versus v* (Figure 2b) were used to determine the exact nature of the oxidation mechanism. According to the Randles-Sevick equation, the plot of anodic peak current (*i_pa_*) *versus v*^1/2^ should be linear for a diffusion controlled process. However, a regression coefficient value was 0.960 from the Figure 2a. It demonstrated the system not a diffusion controlled process. The double logarithmic plot of the anodic peak current (*i_pa_*) *versus v* was further investigated. The slope value of 0.86 was obtained for the CoPc/SPE mediated sulfur oxidation. The slope value of the double logarithmic plot lies in the range from 0.5–1.0 for the mixed adsorption and diffusion controlled process [28]. This result proves that a mixed adsorption and diffusion controlled mechanism was involved in the sulfur oxidation reaction at the CoPc/SPE.

**Figure 3:**
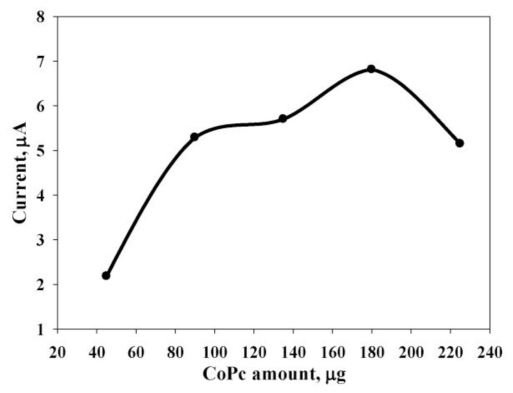
Varying amounts of the CoPc on the electrode surface with 64 mg·L^−1^ of sulfur in a 0.01 mol·L^−1^ NaOH solution at sweeping potential −0.2 V to 0.6 V, *v* = 50 mV·s^−1^.

The catalytic activity of the CoPc/SPE was tested for a sulfur oxidation reaction in the presence of varying amounts of CoPc between 40 μg to 220 μg, as shown in Figure 3. The 180 μg contents of CoPc/SPE provided optimal conditions for sulfur oxidation reaction, as further indicated using flow injection analysis.

### 2.2. Flow Injection Analysis

**Figure 4:**
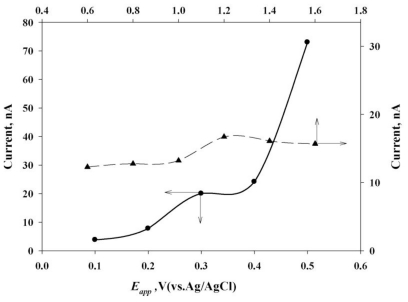
Typical plots of various *E_app_* and *H_f_* with 64 mg·L^−1^ sulfur in a 0.01 mol·L^−1^ NaOH solution, and the sample loop was 20 μL.

The analytical response of the CoPc towards the sulfur oxidation reaction was explored further using FIA coupled with a wall-jet electrode system, to increase sensitivity. Using this method the selective determining of sulfur in cosmetics in the presence of other ingredients is feasible. An initial optimization experiment was conducted for 64 mg·L^−1^ of sulfur under various working parameters, such as applied potential (*E_app_*), flow rates (*H_f_*), and various NaOH concentrations. The effect of applied potential (*E_app_*) was examined by fixing *H_f_* = 1.2 mL·min^−1^, and an optimized response was obtained by increasing the potential from 0.1 V to 0.5 V (*versus* Ag/AgCl), as shown in Figure 4.

**Figure 5:**
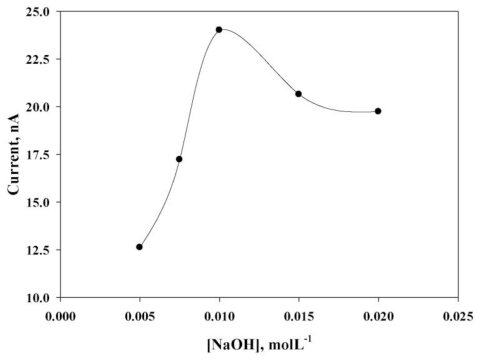
Typical plot of various electrolyte concentration, the *E_app_* = 0.3 V and *H_f_* = 0.3 mL·min^−1^, other conditions are the same as Figure 4.

This study noted that the sulfur detection current increased with the increase in applied potential (*E_app_*). Beyond this potential (0.3 V), the current was unstable due to the oxygen evolution reaction activated at 0.3 V, which may complicate the sulfur oxidation reaction by reflecting a poor stability of 3.95% at 0.3 V, 8.16% at 0.4 V and 10.92% at 0.5 V. The hydrodynamic parameters of the flow rate (*H_f_*) were then optimized using a detection potential of 0.3 V, as suggested in earlier studies. Considering the enhanced peak current and R.S.D value in analytical application, a uniform *H_f_* of 1.2 mL·min^−1^ was chosen for additional FIA experiments. A lower FIA detecting current signal was also observed before and after a *H_f_* of 1.2 mL·min^−1^. These observations indicate that the mechanistic pathways can compete with each other within the working *H_f_* window, and one of the favorable mechanisms may help to achieve the maximum current value of 0.3 V. Additionally, the effect of a NaOH (0.005–0.020 mol·L^−1^) concentration on the sulfur oxidation reaction was also studied, as shown in Figure 5. At higher concentrations of NaOH, the current was reduced when the oxygen revolution reaction at the CoPc/SPE could affect the sulfur oxidation mechanism. The optimized conditions of *H_f_* = 1.2 mL·min^−1^, *E_app_* = 0.3 V and 0.010 mol·L^−1^ NaOH electrolytes were used in subsequent FIA studies.

**Figure 6:**
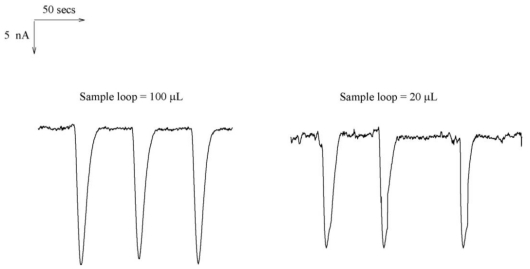
The actual current responses of 100 μL and 20 μL sample loops, other conditions are the same as Figure 5.

For a standard sample of 64 mg·L^−1^ sulfur at 0.01 mol·L^−1^ NaOH, two loops with loop volumes of 100 μL and 20 μL have been applied under the flow rate of 1.2 mL·min^−1^ and a applied potential of 0.3 V (*versus* Ag/AgCl). Their response currents are 19 nA and 16 nA, respectively. Results are shown in Figure 6.

**Figure 7:**
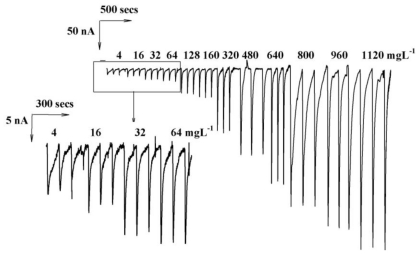
Actual current responses of various concentrations of sulfur. Other conditions same as Figure 6.

No obvious discrepancy in sensitivity between both loop volumes happens. When the sample loop volume is increased, both the peak width and analytical time are increased. Therefore, 20 μL is a suitable loop volume for this study. Figure 7 displays the typical FIA responses observed from 4 to 1120 mg·L^−1^ under optimized conditions. The linear concentration measured up to 1120 mg·L^−1^, with a linear equation and regression coefficient of Y = 8.7316 + 0.272X and 0.9985, respectively (Figure 7). Twelve continuous injections of 64 and 480 mg·L^−1^ sulfur resulted in an R.S.D of 3.60% and 3.52% indicating the detection limit (D_L_, S/N = 3) of 0.325 mg·L^−1^. Overall, the system reproducibility and stability are excellent and disposable electrodes can be used for bulk preparation and routine analytical assays.

### 2.3. Practical Application

The analytical utility of CoPc was also studied in recovery experiments using a commercial anti-acne cream. The recovery of anti-acne cream was established using a standard addition method (see Table 1 for recovery data). The labeled biosulfur fluids values were 5.00% (#1) and 6.30 % (#2) (w/w), and the actual sulfur amounts were 0.08% and 0.101%. The results of the proposed method were 0.078% and 0.101%, and the calculated amounts of biosulfur fluid were 4.88% (#1) and 6.31% (#2), close to the labeled values. In this study, a photometric method was used to detect sulfur amounts in the same cream. The results from the photometric method showed the sulfur values were 0.081% and 0.102% in two anti-acne creams, and the calculated biosulfur fluid amounts were 5.08% and 6.39%. Results from the photometric method (0.081% and 0.102%) were similar to those from the proposed method (0.078% and 0.101%). The recovery rates of the spiked sample ranged between 96.85% and 104.52% (*n* = 3), as shown in Table 1. These results suggest that a CoPc/SPE electrode coupled with flow injection analysis is adequately reliable and sensitive to determine sulfur in commercial anti-acne creams

## 3. Experimental

### 3.1. Materials and Instruments

**Figure 8:**
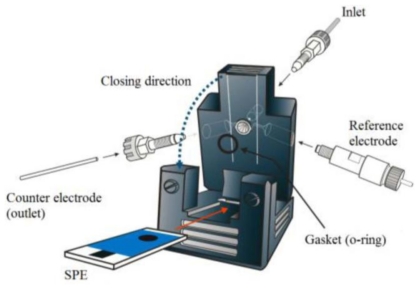
Illustration of the flow cell.

Cobalt phthalocyanine (β-form, Sigma-Aldrich, USA), sodium hydroxide (Showa, Japan), and biosulphur fluid (1.6% (w/w) sulfur and 98.4% of Tween 80 and water, CLR, Germany) were used as obtained without any further purification. All the reagents were of analytical reagent grade and prepared using de-ionized (D.I.) water (18.3 MΩ). Cosmetic cream samples were purchased from local supermarket and homemade. Cyclic voltammetric (CV) measurements were conducted using a CHI800c electrochemical workstation (CHI TX, USA) with a conventional 10 mL working cell. The sulfur photometry was performed using a V-630 photospectrometer (JASCO, Japan). The SPE (SE100-HD) was obtained from Zensor (Taichung, Taiwan). The working area was 3 mm in diameter. The three electrode system consisted of the CoPc/SPE working electrode, an Ag/AgCl (3 mol L^−1^ KCl) reference electrode (BAS RE-5), and a platinum wire auxiliary electrode. The flow cell was obtained from Zensor (Taichung, Taiwan); the construction of the flow cell is displayed in Figure 8, including an Ag/AgCl (3 mol L^−1^ KCl) reference electrode, the outlet (stainless tube) as a counter electrode, and the flow rate controlled by a Master Flex peristaltic pump (Cole Parmer Instrument Company, USA).

### 3.2. Electrode Preparation

The CoPc/SPE modified electrode was prepared by mixing carbon ink and CoPc [40%:60% (w/w)] in 10 times a 1:1 volume ratio of dichloromethane and acetonitrile before. The mixture was drop coated using 5 μL onto the SPE and dried in an oven at 60 ± 2 °C for 5 min. The surface amount of the CoPc was 180 μg.

### 3.3. Flow Injection Analysis (Figure 8)

Flow injection analysis (FIA) was performed using a Rheodyne 7125 sample injection valve (20 μL loop) and equilibrated in a 0.01 mol·L^−1^ NaOH carrier solution at +0.30 V *versus* Ag/AgCl until the current became constant, approximately 5 min at room temperature. The sulfur was quantified by measuring the oxidation peak current in FIA at room temperature (25 °C).

### 3.4. Preparation of Sample Solutions

The anti-acne cream formulated with 5.00% and 6.30% (w/w) biosulfur fluid (0.08% and 0.10% sulfur) was weighed to four digits after the decimal point and dissolved in 100 mL of supporting electrolyte. Next, the mixture was sonicated in an ultrasonic bath for 10 min and filtered sequentially through 0.22 μm critical syringe filters (Critical Process Filtration Inc., Nashua, NH, USA) to remove the suspended colloids. Finally, the filtered solution was diluted to the required concentration for FIA.

### 3.5. Determination of Sulfur by Photometry

The phorometric method, performed according to Wang’s research [20], transferred the elemental sulfur to sulfides and detected sulfides at a wavelength of 262 nm. The absorbing solution (SAOB) for sulfur detection comprised 50 mL of 1 mol·L^−1^ NaOH, 2 g of ascorbic acid and 0.5 g of disodium EDTA. Suitable amounts of the creams to be studied, were weighed accurately in a 50-mL beaker, diluted with approximately 20 mL of cyclohexane and hydrazium hydroxide, dissolved, and filtered into a 25-mL volumetric flask, and the volume increased using cyclohexane and SAOB.

## 4. Conclusions

A simple and inexpensive method for determining sulfur in cosmetics using a CoPc/SPE coupled with flow injection analysis has been established. Analytical characterization of the CoPc/SPE demonstrates an excellent detection limit of 0.32 mg·L^−1^. Additionally, this study demonstrates real sample analysis, without any tedious pretreatment procedures. Since this approach is inexpensive and easily conducted, it can be applied as a convenient portable sulfur analyzer.

## Figures and Tables

**Table 1:** The recovery table of sulfur determination in two anti-acne creams.

Sample *	Original Level Value after Dilution (mg L^−1^) *	Original Detected Value (mg L^−1^)	Spiked Value (mg L^−1^)	Detected Value after Spiked (mg L^−1^)	Recovery (%)
#1 **	12.84	12.48 ± 1.91	16	29.20 ± 0.34	104.52 ± 2.10

			32	43.47 ± 1.68	96.85 ± 5.24

			64	79.62 ± 0.33	104.93 ± 0.52

#2 **	16.16	16.28 ± 0.75	16	31.99 ± 0.27	98.24 ± 1.69

			32	49.49 ± 0.89	103.80 ± 2.77

			64	81.37 ± 2.32	101.71 ± 3.62

* Dilution factor are 62.4 (#1) and 62.8 (#2);

** Corresponding detected values of sulfur after dilution factor correction are 0.078% (#1) and 0.101% (#2).
